# An All-in-One Platform for On-Site Multiplex Foodborne Pathogen Detection Based on Channel-Digital Hybrid Microfluidics

**DOI:** 10.3390/bios14010050

**Published:** 2024-01-18

**Authors:** Mei Xie, Tianlan Chen, Zongwei Cai, Bo Lei, Cheng Dong

**Affiliations:** 1 Department of Life Sciences, Beijing Normal University-Hong Kong Baptist University United International College, Zhuhai 519000, China; 18482864@life.hkbu.edu.hk; 2 Department of Chemistry, Hong Kong Baptist University, Hong Kong SAR, China; 3 Digifluidic Biotech Ltd., Zhuhai 519000, China; 4 School of Intelligent Systems Science and Engineering, Jinan University, Zhuhai 519000, China; 5 Department of Biomedical Engineering, Jinan University, Guangzhou 510632, China

**Keywords:** channel-digital microdevice, full integration, pathogen detection, visually detectable, point-of-care testing

## Abstract

Recently, significant progress has been made in the development of microdevices for point-of-care infectious disease detection. However, most microdevices only allow limited steps, such as DNA amplification on the chip, while sample preparation, such as lysis and DNA extraction, is conducted off the chip using the traditional method. In this study, an all-in-one platform was developed, which incorporated all necessary procedures for nucleic acid detection. Our on-chip DNA extraction method utilized the magnetic bead-based technology on a hybrid channel-digital microfluidics (C-DMF) microdevice. It yielded high recovery rates, varying from 88.43% to 95.83%, with pathogen concentrations of 10^3^–10^6^ CFU/mL. In particular, the on-chip method exhibited significantly higher efficacy compared to the traditional off-chip manual method, for the DNA extraction of *E. coli* and *S. aureus*, representing Gram-negative and Gram-positive bacteria, respectively, at a sample concentration of 10^3^ CFU/mL. To address the need for rapid and accessible diagnostics, colorimetric LAMP amplification was integrated into the proposed microdevice. The results were visually detectable with the naked eye, making it user-friendly for non-specialists. In addition, this platform demonstrated impressive sensitivity in simultaneously detecting common foodborne pathogens in spiked meat samples, achieving the LOD of 10^2^–10^3^ CFU/mL. The entire process, from sampling to result, was fully automated and only required approximately 60 min, offering promising applicability in resource-limited and on-site testing scenarios.

## 1. Introduction

Pathogenic microorganisms have caused a variety of infectious diseases that pose a significant threat to public health and the economy worldwide. As reported by the World Health Organization (WHO), approximately 600 million people worldwide suffer from foodborne illnesses after consuming contaminated food every year, resulting in an estimated 420,000 deaths [1]. The cost of these illnesses can be substantial, with the expense of medical treatment and lost productivity estimated to be billions of dollars each year [2,3]. Timely identification of pathogenic microorganisms in food is crucial to reduce the risk of illness and improve food safety. The development of novel methods for the rapid and accurate detection of foodborne pathogens, thereby, is of immense importance.

Point-of-care testing (POCT) has flourished in medical diagnostics for swiftly detecting and monitoring infectious diseases in recent years [4]. In the realm of food safety, POCT has gained significant popularity in the detection of foodborne pathogens, which enables convenient on-site sample handling and delivers rapid results directly at the sampling site, making it especially advantageous in settings with limited resources [5]. To date, nucleic acid testing (NAT) systems have showcased notable advancements through two prominent microfluidic platforms, digital microfluidics and continuous flow microfluidics [6,7,8]. Considerable efforts have been invested in creating fully integrated platforms that incorporate all essential components for nucleic acid testing [6,9,10,11]. An exemplary example of the continuous flow microdevice is the centrifugal microdevice [12,13]. The centrifugal force was employed to control the fluid flow in the microdevice and dedicated channel networks and mechanical valves were utilized to achieve precise volume, efficient mixing, separation, and reagent transportation. Hence, the integration of numerous procedures would introduce complexity and difficulties to the manufacturing process. In contrast, digital microfluidic devices employ an electrode array to individually manipulate droplets on a flat surface, providing advantages in terms of flexibility and compatibility [14]. For example, the different reagents could be pre-dehydrated on any sites of the bottom plate and individual droplets could be manipulated to transport, mix, merge, and split on the plate through any pathway on electrode arrays via EWOD force, with no need for microchannel structure and external driven forces. The unique features of manipulating individual droplets via electrowetting-on-dielectric (EWOD) and programmability make it particularly suitable for implementing complex protocols with no requirement for sophisticated chip architectures.

Most existing DMF microdevices only incorporate partial steps on the chip while sample preparation tasks like cell lysis, DNA extraction, and purification are still performed off the chip using traditional methods [15,16,17,18,19,20] or coupled with another sample preparation device [3,21,22,23,24,25]. Recently, innovations have been focused on incorporating the entire process, such as DNA extraction, nucleic acid amplification, and detection, into a compact device [14,26,27]. The integrated platforms for nucleic acid testing provided promising avenues for efficient and streamlined molecular diagnostics. However, there are several major challenges to the development of the all-in-one platforms with a typical DMF chip. First, with a typical two-plate structure, the capacity of a DMF chip is very limited. Consequently, the microfluidic chips can only accommodate very small sample volumes, which may lead to low sensitivity without pre-enrichment steps. Second, the channel-free architecture of the DMF chip poses challenges in pre-storing liquid reagents, such as washing and elution buffers, due to the potential for droplet displacement during processing and transportation while loading reagents right before the experiment introduces additional manual steps and increases the risks of contamination.

To address the limitations, here we established a channel-digital microfluidics (C-DMF) hybrid platform that consists of a channel structure for magnetic bead-based DNA extraction and a DMF chip structure for parallel LAMP reactions. The platform allowed extraction from milliliter-scale samples to microliter-scale output in a single device, with desirable DNA recovery rates. The typical structure DMF chip was modified with a cover slide so that liquid reagents for DNA extraction were able to be pre-stored at the specified sites. Meanwhile, the LAMP reagents and dye were dried on the chip. In this way, the integrated platform encompassed all the necessary elements for on-chip DNA extraction and nucleic acid testing, enabling the automation of the entire process from sample to result. Furthermore, colorimetric LAMP with neutral red was optimized for direct visual detection with the naked eye. The practicality of the proposed platform was verified by the detection of various pathogenic bacteria in spiked meat samples, highlighting its detection efficiency and sample-to-answer capability.

## 2. Materials and Methods

### 2.1. Materials

All primers and plasmids were synthesized by Sangon Biotech (Shanghai, China); (NH4)_2_SO_4_, KCl, KOH, Tween 20, and neutral red were purchased from Sigma-Aldrich (Shanghai, China). Deoxynucleotide triphosphates (dNTPs) and MgSO_4_ were obtained from New England Biolabs Ltd. (Beijing, China). Glycerol-Free Bst polymerase (MDX018-08A) was purchased from Meridian Biotech Ltd. (Shenzhen, China); the DNA extraction kit (D7113) was purchased from Magen Biotech Ltd. (Guangzhou, China). DMF chips were provided by Digifluidic Ltd. (Zhuhai, China).

### 2.2. Platform Overview and Assay Mechanism

**Figure 1:**
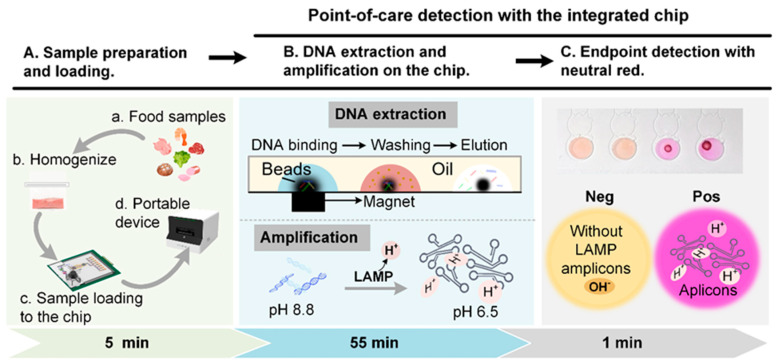
Schematic overview of this study. (**A**). The workflow from sample preparation to the result. (**B**). The mechanisms of the DNA extraction and colorimetric LAMP on the integrated microdevice. (**C**). The result of the colorimetric LAMP reaction with neutral red as the indicator.

The testing procedures of pathogenic bacteria in food samples are presented in Figure 1A. Firstly, the sample was collected in an aseptic sampling bag and homogenized with sterile saline. The aliquot of homogenate was mixed with the lysis buffer and magnetic beads. The mixture was then loaded into the chip and plugged into a portable control device (Digifluidic, Guangdong, China). The integrated platform combined all steps of magnetic bead-based DNA extraction, parallel LAMP reaction, and endpoint visual detection into a hybrid chip, which enabled the fully automated POCT diagnosis of pathogens, with no need for additional manual steps.

The mechanism of LAMP detection was based on the pH-sensitive characteristic of neutral red. The initial pH value of the LAMP reaction solution was adjusted to 8.8. With the existence of target DNA, the templates were replicated and released hydrogen ions, resulting in a pH drop of about 2 units [28]. Under the low-buffering condition, the pH of positive amplification decreased to 6.0–6.5 while the solution of the negative reaction remained alkaline. Neutral red, as the colorimetric indicator, shows yellow in the alkaline and red in the acidic. Therefore, the color could indicate the results of LAMP reactions where red represents positive reactions and yellow negative ones (Figure 1C). The result was directly read with the naked eye.

### 2.3. Design and Fabrication of the Hybrid Chip

**Figure 2:**
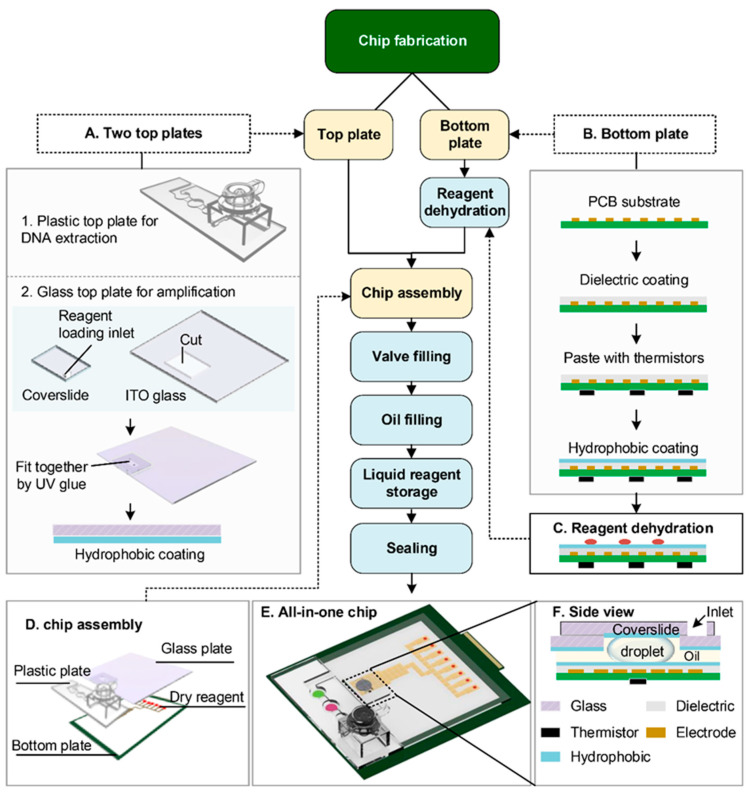
Illustration of chip fabrication. (**A**) The structure and processing of two top plates. 1. Illustration of the plastic top plate. 2. Illustration of the glass top plate. (**B**) The fabrication procedures of the bottom plate. (**C**) Reaction distribution and dehydration on the bottom plate. (**D**) An exploded view of the chip components, which includes a PCB bottom plate with dehydrated reagents, the top plate of the extraction region, and the top plate of the amplification region. (**E**) The image of the all-in-one chip, which encompasses all elements for DNA extraction and testing. (**F**) The side view of the region with a coverslide shows the structure that confines a droplet in the designated location.

The chip consists of a PCB bottom plate and hybrid top plates. As shown in Figure 2A, the DNA extraction region was shielded by a 3-D printed plastic top plate (Figure 2A(1) while the nucleic acid elution region was covered by a modified ITO-glass plate (Figure 2A(2)). The plastic plate was designed with a chamber for both cell lysis and DNA enrichment, which enabled the processing of a large volume of the sample. Following the cell lysis chamber, there were three washing chambers for DNA purification.

The amplification area of the DMF chip has a standard parallel plate structure, where the top plate is made of ITO glass with slight modifications. The intact ITO glass was cut out of a rectangular area (5 mm × 12 mm) and covered with a cover slide instead. As shown in Figure 2A(2), the coverslide and ITO glass were then stuck together with UV glue (Zhuolide 5604, Foshan, China). As illustrated in Figure 2F, the cut section of ITO glass and the coverslide formed a configuration that kept the droplet securely in place between the coverslip and the bottom plate, even during transportation, unless applying EWOD force. This configuration enabled the pre-storage of liquid reagents in the DMF chip.

The PCB bottom plate patterned with electrode arrays was processed as shown in Figure 2B. Initially, a multilayered polyimide dielectric film was applied to the surface through hot pressing and thermistors were affixed on the opposite side as temperature sensors. The thermistors were located at the center of each heating region. To facilitate droplet actuation, the bottom plate was coated with FluoroPel, creating a hydrophobic surface. The parameters were the same as those marked in our previous study [29]. LAMP reagents were distributed to the reaction sites and dehydrated on the surface of the bottom plate (Figure 2C). The chip was then assembled as shown in Figure 2D; the plastic plate was first glued up with the bottom plate and, then, the glass top plate was attached to the bottom plate with the double-side adhesive of 0.6 mm thickness. The two plates were jointed and sealed with UV glue. After chip assembly, oil medium, washing, and elution solutions were sequentially injected into the chip through the loading inlets on the top plates. Finally, the chip was completely sealed and vacuum-packaged. Figure 2E shows the image of the all-in-one chip.

### 2.4. Overview of the Microfluidic System and Workflow on the Chip

**Figure 3:**
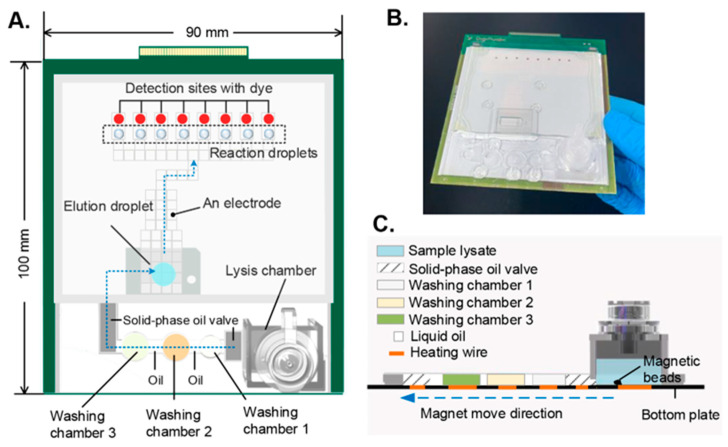
Overview of the microfluidic system. (**A**) The layout of the integrated chip. (**B**) Photograph of the chip. (**C**) Side view of the extraction part.

The chip layout is depicted in Figure 3A. First, the sample was loaded into the lysis chamber and incubated with lysis buffer and magnetic beads for cell lysis and DNA enrichment. Then, the valve between the lysis chamber and Washing Chamber 1 was heated to melt and the magnetic bead carrying DNA was transported to Washing Chamber 1 with the help of an external magnet positioned beneath. Next, the magnetic beads went through several washing cycles in Washing Chamber 2 and Washing Chamber 3, sequentially, to remove impurities. The purified DNA was finally eluted in the pre-stored elution droplet. After DNA extraction, the eluted droplet was split into several smaller droplets (5 μL) and distributed to the parallel reaction sites. The dehydrated reagents on the reaction sites were reconstituted once the droplet arrived. On-chip LAMP was performed at 62 °C for 30 min. After amplification, the droplets were actuated to the detection sites and the results were directly read with the naked eye based on the color of the dye. The photograph of the integrated chip is presented in Figure 3B. The side view shows the way that the lysis chamber connects with the washing chambers (Figure 3C). The solid-oil valve was used to prevent the oil and pre-stored solution from leaking into the lysis chamber during transportation in the case of vehicle turbulence. Once extraction was finished, the solid-phase oil was heated to the liquid phase so that the magnetic beads were allowed to pass through and reach Washing Chamber 1. After that, the valve turned into its solid state within a short time when heating was stopped. Therefore, the solid–liquid phase transition of the oil could work as a valve to control the connecting and closing of the channel between the extraction chamber and the cleaning chamber.

**Figure 4:**
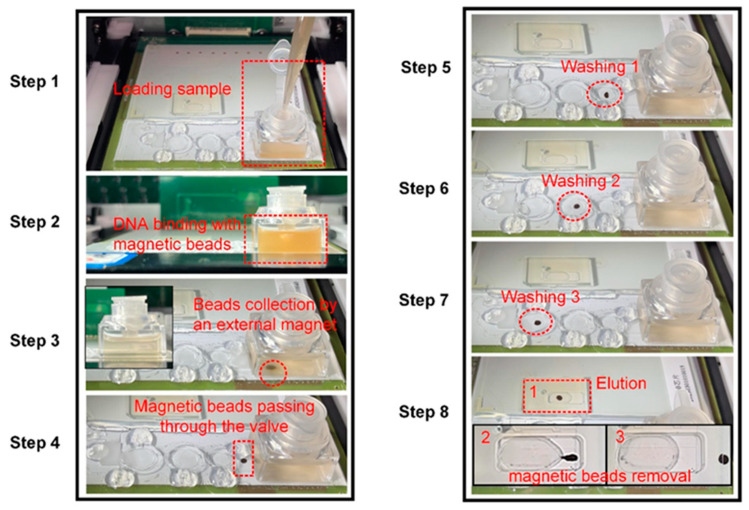
The diagram flow of on-chip DNA extraction.

The on-chip DNA extraction procedures are illustrated in Figure 4. Firstly, an aliquot of 1 mL of the sample was mixed with 1 mL lysis buffer, 20 μL proteinase K (50 μg/mL), and 15 μL magnetic beads (300 nm, 25 mg/mL) before loading into the lysis chamber (Step 1). The mixture was incubated at 75 °C for 10 min for cell lysis and the released DNA was captured by the magnetic beads (Step 2). After DNA enrichment, the magnetic beads were collected by an external magnet underneath the chip (Step 3). It was apparent that the suspension became clarified, which indicated good recovery of the magnetic beads. Then, the valve connecting the lysis chamber and Washing Chamber 1 was heated to the liquid phase and magnetic beads carrying DNA were transported to Washing Chamber 1 (Step 4). The washing chamber filled with oil was heated to 55 °C for 1 min to remove certain oil-soluble impurities (Step 5) and the magnetic beads were then driven to Washing Chamber 2 (40% PEG 6000, 2 M GuCl) and Washing Chamber 3 (40% PEG 6000, 100 mM NaCl, 10 mM Tris-HCl), sequentially, and mixed with the buffer for certain cycles to remove proteins, salts, and other contaminants from the sample (Step 5 and Step 6). PEG plays a crucial role in the DNA-washing process. Under a certain salt concentration, PEG induces conformational changes in DNA, thus enhancing nucleic acid-magnetic bead affinity. It can also increase the viscosity of the system, thereby ensuring the sustained suspension of magnetic beads. In addition, PEG is conducive to removing carbohydrate and protein residuals from the sample [30,31]. At last, the purified DNA was eluted in a 50 μL droplet (0.1% Tween 20, DNase-free water, pH 8.8) and the magnetic beads were moved away from the DNA elution (Step 8). The motion of the magnetic beads was manipulated with an external magnet held by a stepping motor mounted on the device, which was controlled with a customized program.

### 2.5. Regents Dehydration and Colorimetric LAMP

The components for the LAMP reaction were divided into two parts; a mixture containing dNTPs, primers, and the glycerol-free Bst polymerase were prepared in a 5 × solution. To eliminate any negative effect of salts on the enzyme activity during dehydration, components including (NH4)_2_SO_4_, KCl, and MgSO_4_ were prepared in another mix and dried on the pathway of the droplet transportation. The detection sites were loaded with 1.5 μL neutral red (2.5 mM). The two mixtures and dye were added with 1% excipient and dried at 37 °C for 20 min under a flow of clean air. The final formula for the LAMP reaction was 8 mM MgSO_4_, 10 mM (NH4)_2_SO_4_, 50 mM KCl, 8 U glycerol-free Bst polymerase, 1.4 mM dNTPs, 1.6 μM (each) of the inner primers, 0.8 μM (each) of the loop primers, and 0.2 μM (each) of the outer primers. Bacterial strains and primers were prepared as described in our previous study [19].

### 2.6. Evaluation of Sensitivity

The pure culture of each strain was collected and counted by the standard plate counting method. The cell suspension of all the bacteria was diluted to 10^6^ CFU/mL. A mixed sample solution was prepared by adding 1 mL of each bacteria solution into sterile saline, giving a final concentration of 10^5^ CFU/mL of each target. Serial dilutions were prepared similarly. The dilutions with concentrations ranging from 10^1^ to 10^5^ CFU/mL were subjected to the proposed microfluidic platform for on-chip extraction and then dispensed into droplets of 5 μL for the LAMP reaction and subsequent visual detection.

### 2.7. Verification of the All-in-One DMF System by the Detection of Multiple Pathogens in Spiked Food Samples

The feasibility of the lab-on-a-chip (LOC) system for rapid and parallel detection of multiple foodborne pathogens was demonstrated using spiked meat samples. For each kind of pathogenic bacteria, the number of CFU per mL in the fresh culture was enumerated by the standard plate counting method and diluted to a concentration of 10^6^ CFU/mL with sterile saline. Fresh pork meat, locally purchased, was autoclaved to ensure that it was free of any target pathogens and this was confirmed by qPCR tests. The meat sample was aseptically minced before being collected in an aseptic sampling bag and homogenized with saline (1:5 *w*/*v*) by hand for about 2 min. Aliquots of the 6 mL sample homogenate were inoculated with a 1 mL diluted solution of each bacterial species, resulting in a 10 mL mixed sample with a concentration of 10^5^ CFU/mL for each microorganism. The sample was further diluted to 10^4^–10^1^ CFU/mL and loaded into the LOC system. Meanwhile, the non-spiked samples were employed as the negative control.

## 3. Results and Discussion

### 3.1. Optimization of the Extraction Conditions

**Figure 5:**
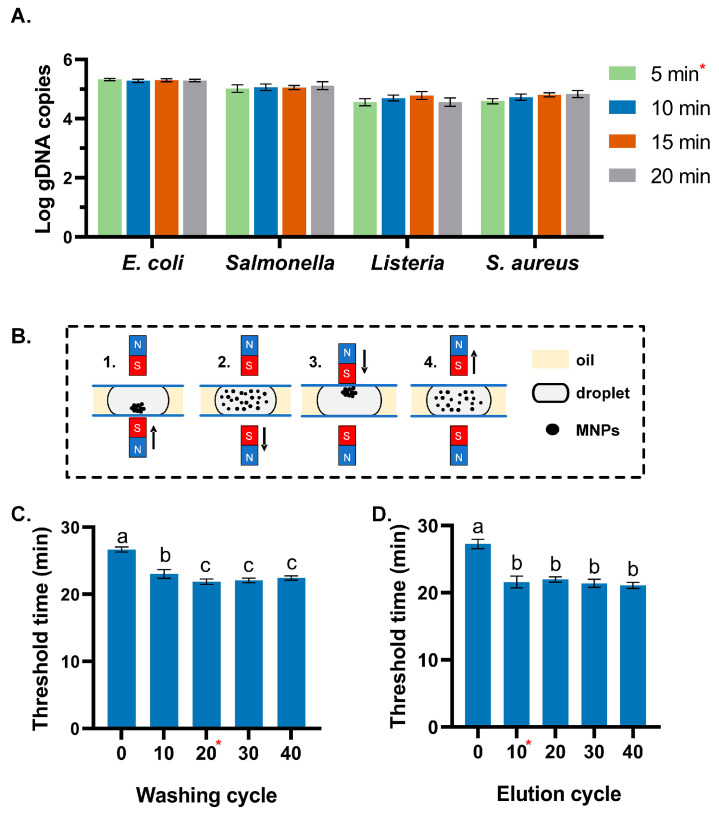
Optimization of the on-chip DNA extraction conditions. (**A**) Cell lysis at 65 °C for different incubation times. (**B**) Illustration of a mixing cycle during the washing and elution process. (**C**) The threshold time of LAMP with the DNA extracted by washing with different mixing cycles. (**D**) The threshold time of LAMP with the DNA extracted with the elution of different mixing cycles. The optimum conditions of this study are highlighted with a mark of a subscript red asterisk (*). The significance is marked with letters. For variables with the same letter, the difference between the means is statistically insignificant.

An ideal DNA extraction profile should maximize the DNA releases during cell lysis and minimize DNA loss during purification steps, such as binding and washing, while ensuring a high DNA yield during elution. Here, the optimal duration for cell lysis was determined based on the DNA yield under various conditions. Initially, bacterial cells from four microorganisms were harvested from the culture medium and subsequently suspended in a TE buffer. An aliquot of the 1 mL cell suspension, with a random concentration, was utilized for the lysis experiments. The Gram-positive bacteria were incubated with lysozyme (20 mg/mL) at 37 °C for 10 min before further processing. As the proteinase K used in this study exhibits best activity at 65 °C, the temperature was set at 65 °C and the duration time was optimized at 5 min, 10 min, 15 min, and 20 min, respectively. After purification, the recovered DNA was quantified by the qPCR method. Each experiment was tested in triplicates. The result was shown in Figure 5A; no significant difference was found regarding the variation of incubation time, which indicated sufficient lysis at 65 °C for 5 min.

Purification is crucial for assuring the quality and yield of DNA. Here, the mixing cycle of magnetic beads during the washing and elution process was optimized. The sample was prepared by adding 1 mL *S. aureus* (10^7^ CFU/mL) to 9 mL meat homogenate. The spiked meat sample was introduced to the chip for DNA extraction. Figure 5B illustrates a complete mixing cycle for DNA washing and elution. A magnet was placed both on top of and beneath the droplet. Once the magnetic beads reached the washing/elution chamber, the magnet attached to the bottom plate was lowered. Without the magnetic attraction, the magnetic beads were dispersed in the droplet. Subsequently, the upper magnet moved down to gather the magnetic beads within the droplet and, then, released them into the droplet once more. The use of an upper magnet could help prevent some magnetic beads from gathering at the bottom of the chip and improve the contact efficiency between magnetic beads and liquid, thus ensuring adequate DNA purification and elution efficiency. The purified DNA obtained from the chip was subsequently tested using a standard real-time LAMP assay to assess the efficiency of the extraction process, with the threshold time (Tr) serving as a comparative index. The result is shown in Figure 5C; the crude DNA extract without sufficient washing resulted in the largest Tr value (26.7 min), which indicated possible carryover of inhibitors by the magnetic beads or low recovery of DNA from the sample. The fastest reaction was observed under the condition of 20 washing cycles, offering a result within 21 min. Although LAMP was reported to be more tolerant to common inhibitors [32,33,34,35], the result indicated that purification is still necessary to ensure good sensitivity regarding food samples with abundant matrices, such as meat. The optimum condition was obtained with 20 mixing cycles and 10 cycles for the washing and the elution process, respectively (Figure 5C). However, excessive washing could lead to a longer reaction time, which might be caused by an increase in DNA loss during the washing step. As for DNA elution, DNA eluted by mixing the magnetic beads with the dilution buffer for 10 cycles resulted in a significantly higher DNA yield than the non-mixing group, as evidenced by a significant decrease in the threshold time of LAMP reaction from 27.3 min to 21.6 min. The result demonstrated that the efficient mixing of the magnetic beads and the elution buffer greatly improved the extraction efficiency. In this way, a mixing cycle took only 5 s; thereby, the overall time for the DNA extraction on the chip was about 10 min under the optimized condition, which is more efficient than the standard laboratory method and the previously reported microfluidic-based assays [36,37,38,39].

### 3.2. Evaluation of the Extraction Efficiency

**Figure 6:**
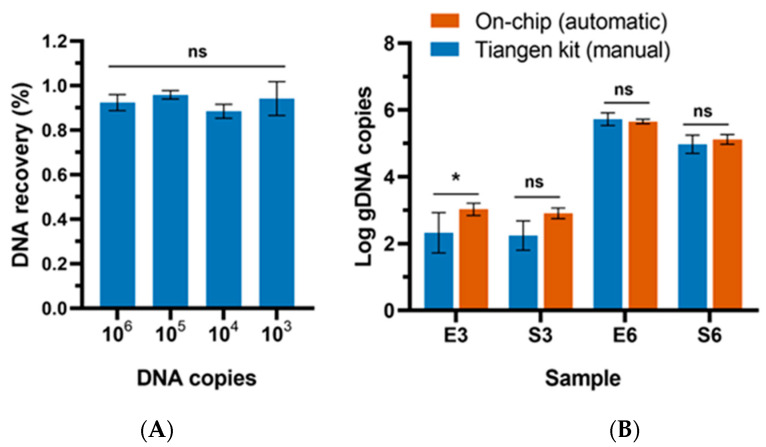
The extraction efficiency of this method. (**A**) The DNA recovery rates of different input gDNA concentrations extracted using the proposed microdevice. **(B**) A comparison of the on-chip extraction method with the traditional DNA extraction method using a magnetic-based DNA extraction kit. E3: 10^3^ CFU/mL of *E. coli*; S3: 10^3^ CFU/mL of *S. aureus*; E6: 10^6^ CFU/mL of *E. coli*; S6: 10^6^ CFU/mL of *S. aureus*. Significance is marked with “*” (*p* < 0.05); ns represents no significance.

To evaluate the extraction capability of this device, the gDNA of *E. coli* of known copies was diluted ten-fold in series. The dilutions with the final concentrations of 10^3^–10^6^ copies/mL were directly used as the standard sample for on-chip DNA extraction. The DNA elution was collected after the extraction process and quantified by the qPCR method. As shown in Figure 6A, the recovery rate of this method varied from 88.43% to 95.83%, depending on the input gDNA concentrations. From previous assays using microdevices for DNA extraction with similar spiked levels, the best recovery rate ranged from 73% to 91.3% [40,41,42]. In comparison, the recovery rate of this study is higher. It is important to highlight that this platform exhibited superior performance when tested with low input concentrations with a high recovery rate of up to 94.17%, which indicated a significant potential for early screening of contamination.

To further evaluate the effectiveness of this platform in extracting DNA from bacteria cells across different cell densities, on-chip tests were performed using samples diluted to concentrations of 10^6^ CFU/mL and 10^3^ CFU/mL. *E. coli* and *S. aureus* were selected as the Gram-negative and Gram-positive representatives. For comparison, a conventional off-chip assay was conducted simultaneously using a commercial magnetic bead-based DNA extraction kit (Tiangen, DP705, Beijing, China) following the manufacturer’s instructions. Briefly, an aliquot of 1 mL of bacterial culture was centrifuged and the bacterial pellets were collected by centrifugation at 10,000 rpm for 1 min. The pellets were then added with lysis buffer and incubated at 75 °C for 15 min. After cell lysis, magnetic beads were added to the lysate for DNA enrichment. The mixture was set at room temperature for 9 min, with interval shaking for 1 min every 3 min. The magnetic beads carrying DNA were then manually washed twice on a magnetic grate to remove the impurities. Finally, DNA was released into the elution buffer.

The result indicated that the on-chip assay outperformed the off-chip kit method in terms of processing low-concentration samples (Figure 6B). The recovery of DNA extracted from 10^3^ CFU/mL of *E. coli* cell suspension by the on-chip method was significantly greater than that obtained by the kit (*p* < 0.05). There are two main factors contributing to the outcome. Firstly, the reduction in the amount of buffer utilized during the washing steps effectively minimized DNA loss [43]. Secondly, the utilization of an automated microscale extraction process with the microdevice proved advantageous in collecting magnetic beads from the suspension, thereby optimizing DNA recovery. Moreover, the mixing mode of on-chip DNA washing and elution based on magnetic beads reduced the unwanted adsorption of DNA analytes. Meanwhile, the traditional mixing method, such as vortex in a tube, was prone to cause DNA adsorption on the tube wall. The DNA loss was particularly pronounced when the template content was low [44].

### 3.3. Sensitivity of Microfluidic-Based LAMP Detection

**Figure 7:**
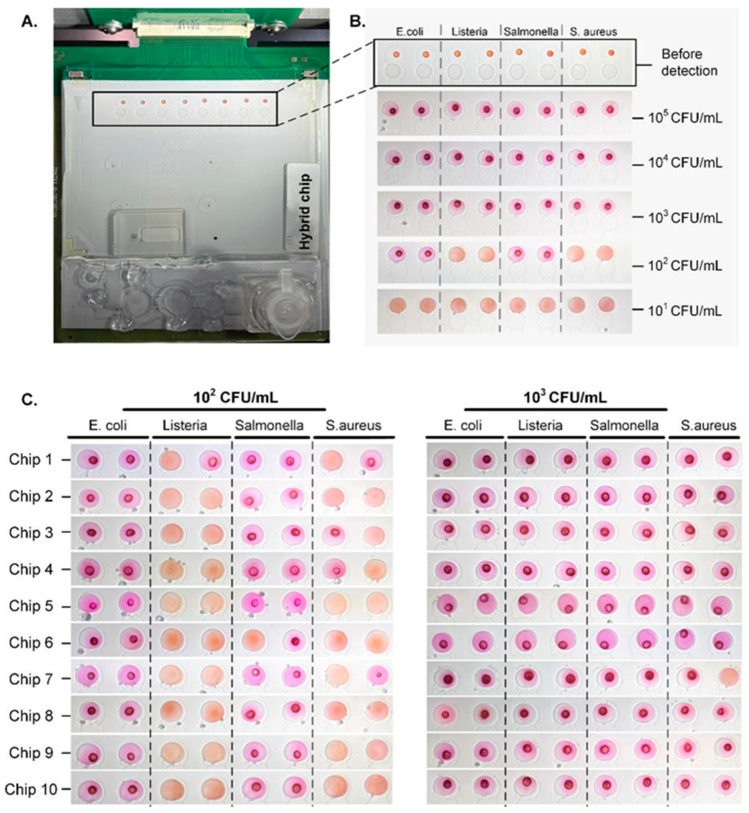
Sensitivity of multiplex detection with the microdevice. (**A**) The digital image of the hybrid chip for nucleic acid extraction and testing. Inset highlights the reaction droplets and detection dye on the chip before colorimetric detection. (**B**) Illustration of the results of the LAMP detection of multiple pathogens at various concentrations. Neutral red was used as the indicator. (**C**) The results of LOD tests with multiple bacteria at the concentrations of 10^2^ CFU/mL and 10^3^ CFU/mL, each with 20 reaction spots.

The sensitivity was determined by multiplex tests on the chip, as shown in Figure 7. The primer sets specifically targeting *E. coli*, *L. monocytogenes*, *S. typhimurium*, and *S. aureus* were, respectively, aligned on the reaction spots, as shown in Figure 7B. Each target was set with two parallel tests. As shown in Figure 7B, samples with concentrations below 10^2^ CFU/mL were not detected in any of the samples. From 10^3^ to 10^5^ CFU/mL, all reactions exhibited a vivid red color, indicating a 100% detection sensitivity. At the concentration of 10^2^ CFU/mL, only *E. coli* and *S. typhimurium* exhibited positive results; whereas, *L. monocytogenes* and *S. aureus* could not be identified.

The limit of detection (LOD) is determined according to the FDA’s emergency use authorization guideline referring to the minimum DNA copy number that can be detected with a frequency of 95% or higher in at least 20 reactions. As shown in Figure 7C, the LOD was 10^2^ CFU/mL for *E. coli* and *S. typhimurium*, with detection rates of 100% (20/20) and 95% (19/20), respectively. While the LOD for *S. aureus* and *L. monocytogenes* was 10^3^ CFU/mL. The system showed higher sensitivity in detecting Gram-negative bacteria compared to Gram-positive bacteria. A potential reason could be that extracting DNA from Gram-positive bacteria (*L. monocytogenes* and *S. aureus*) using this method was likely more challenging compared to Gram-negative bacteria *(S. typhimurium* and *E. coli*). The difference is attributed to the difference in cell wall structure between Gram-positive and Gram-negative bacteria [45]. In Gram-positive bacteria, the cells were protected by a thick multilayer of extensively crosslinked peptidoglycan. In contrast, Gram-negative bacteria have a thinner and simpler cell wall structure. Consequently, methods employed for genome extraction tend to be more effective in Gram-negative bacteria [46].

The endpoint detection process in this study could be completed within 1 min. Within a short time, the neutral red dye was not fully dissolved and the dry reagent residues formed a distinct red circle within the droplet, as depicted in the positive reaction (Figure 7B). On the other hand, in the negative reaction droplet with an alkaline solution, the dye dissolved more rapidly, resulting in a solution without noticeable precipitation. This characteristic helped distinguish positive results from negative ones, in addition to the discernible color difference.

### 3.4. Application of the Integrated Platform for Pathogen Detection in Spiked Meat

To verify the applicability of the assay, the platform was employed for the detection of meat samples spiked with multiple pathogens. As shown in Table 1, the system could occasionally identify as low as 10^2^ CFU/mL of *S. typhimurium* and *E. coli* in the spiked meat samples and 10^3^ CFU/mL of *L. monocytogenes* and *S. aureus*. The detection rates were lower than those using pure cultures. The sensitivity decrease could be attributed to food matrices. Without centrifugal pretreatment, the sample homogenate contains massive tissue impurities, which may hinder the binding of DNA with beads during extraction, resulting in less DNA recovery. Future improvements, such as the integration of portable syringe filtration units for sample pretreatment, are expected to solve this problem [47]. Overall, the sensitivity of this study was comparable with some published integrated POCT platforms. For instance, Tang et al. introduced an integrated paper-based system capable of performing nucleic acid extraction and amplification, which successfully detected *S. typhimurium* in milk samples at concentrations as low as 10^3^ CFU/mL [48]. Chen et al. introduced an integrated multiplex system capable of simultaneously detecting five pathogens in urine samples, with the limits of detection ranging from 10^2^ CFU/mL to 10^3^ CFU/mL [49]. Kim et al. developed a finger-actuated nucleic acid extraction and purification chip, offering a sensitivity to detect as low as 10^3^ CFU/mL of *E. coli* in the blood sample [50].

## 4. Conclusions

This study successfully established a fully integrated platform for the POCT detection of multiple foodborne pathogens. This platform included all necessary procedures of nucleic acid detection, including magnetic bead-based DNA extraction, LAMP amplification, and endpoint detection, into a hybrid-structured chip, which enabled a large volume sample treatment and streamlined the DNA extraction and testing steps on the chip with no need for tedious manual steps or bulky equipment. Combining with colorimetric LAMP assay, the result was visually detectable with the naked eye and accessible to non-specialized users, which enhanced its applicability in resource-limited areas and on-site testing scenarios. The time from sample to result using this platform was about 60 min and the whole process on the chip was fully automated. The detection limit of the multiplex detection of common foodborne pathogens using this platform was 10^2^–10^3^ copies/mL of genomic DNA and 10^3^–10^4^ CFU/mL cells in real food samples. In general, the suggested microfluidic platforms designed for nucleic acid testing offer promising avenues for efficient and streamlined molecular diagnostics. Further improvements should be made to enhance the extraction efficacy from challenging cell types, like Gram-positive bacteria. Additionally, we will continue to extend the platform to a wider range of applications, such as food authentication, medical diagnostics, and so on.

## Figures and Tables

**Table 1:** The result of pathogen detection in artificially contaminated meat with the integrated platform.

Concentration ^a^ (CFU/mL)	Result ^b^			
	*E. coli*	*S. typhimurium*	*L. monocytogenes*	*S. aureus*
10^4^	6/6	6/6	6/6	6/6
10^3^	6/6	6/6	4/6	2/6
10^2^	4/6	3/6	0/6	0/6
10^1^	0/6	0/6	0/6	0/6

^a^ The displayed concentrations indicate the final concentration of each species. ^b^ The result is expressed as the number of positive tests in the total number of tests.

## Data Availability

Data are contained within the article.
